# Changes in self-rated health and its association with social determinants – repeated cross-sectional surveys among Finnish adolescents from 1981 to 2025

**DOI:** 10.1186/s12889-026-27022-y

**Published:** 2026-03-18

**Authors:** Heidi Kesanto-Jokipolvi, Timo Ståhl, Jaana M. Kinnunen, Reija Autio, Risto Hotulainen, Arja Rimpelä

**Affiliations:** 1 Faculty of social sciences, Health Sciences, Tampere University, Arvo Ylpön katu 34, Tampere, 33520 Finland; 2 Finnish Institute Health and Welfare, Promotional and Preventive work, Mannerheimintie 166, Helsinki, Finland; 3 Department of Education, University of Helsinki, Siltavuorenpenger 3A, Helsinki, 00017 Finland; 4 Department of Adolescent Psychiatry, Tampere University Hospital, Elämänaukio 2, Tampere, 33520 Finland

**Keywords:** Self-rated health, Adolescents, Social determinants of health, School environment

## Abstract

**Background:**

There are a lack of a societal perspective and a comprehensive approach to adolescents’ wellbeing and their declining mental health. We study here changes in Finnish adolescents’ self-rated health (SRH) in 1981–2025, and how SRH associated with societal changes, and sociodemographic and school-related factors during these years.

**Methods:**

Two data sets of 14-year-old Finns were used: nationwide postal/internet surveys 1981–2019 (N = 28 960) and school-surveys 2000–2025 (N = 604 758), both conducted biennially. Explanatory variables for low SRH in gender-stratified logistic regression analyses were survey year, sociodemographic factors (gender, parents’ education and smoking, family structure), and school-related factors (teacher relationship, classroom atmosphere, learning difficulties, school performance).

**Results:**

The prevalence of low SRH remained steady in 1981–2015. From 2015 onwards, low SRH started to increase, but only among girls, peaking in 2023. All explanatory variables were associated with SRH, but their changes accounted for 9% of the increase in girls’ low SRH. Minor variations were observed in the associations between SRH and the explaining variables over time, except a strengthened association between low SRH and learning difficulties in 2015–2023.

**Conclusion:**

Before 2015, adolescents’ low SRH remained relatively steady, despite improvements in family sociodemographic indicators, positive changes in school environment, and factors such as the economic recession in the 1990s. From 2015 onwards, the gender gap began to widen, peaking during COVID-19 pandemic, consistent with previous studies on gender differences in mental health. Concurrently, the strong association between low SRH and learning difficulties further strengthened among both genders, despite that known risk factors diminished in families and school environment improved. Further research is necessary to reveal factors that influence adolescents’ wellbeing and learning in contemporary society.

**Supplementary Information:**

The online version contains supplementary material available at 10.1186/s12889-026-27022-y.

## Introduction

Over the past few decades, significant societal changes and global events have shaped living environments and experiences of adolescents, the most recent being the COVID-19 pandemic and the emergence of social media [1]. Our understanding of how various factors have contributed to changes in adolescent health and well-being over time remains incomplete. The mental health crisis, a rather rapid decline in the mental health and increased stress symptoms of adolescents across the Nordic countries [1–4] highlights the need to examine the determinants of health and wellbeing in general and through the lens of social determinants and their evolution over time. However, data spanning several decades are scarce. Self-rated health (SRH) as a standard question in surveys provides an opportunity for long-term assessment of comprehensive wellbeing at the population level using internationally comparable indicator [5].

Self-rated health is a measure of people’s perception of their own health. Already in adolescence, health is conceptualized using social, mental, and physical aspects of their life [6]. In SRH, different aspects of health, socio-cultural context, biological factors, and psychological experiences intersect within the individual-level experience [7, 8]. This study examines adolescents’ low SRH and its variations in response to societal changes over the past four decades. Low SRH at a younger age has been shown to predict health care attendance, medication use, and even mortality in later life [9–11].

The second aim of this study is to investigate changes in the association between SRH and its social determinants over time. An example, increasing emotional problems among adolescents are associated with trends of increased parental emotional problems, family social inequality and school-related stress [12]. However, yet little is known about changes in risk and protective factors of adolescents’ health and wellbeing. Here, we examine family sociodemographic and school-related factors over time in terms of SRH. In our study, family factors and relationships in the school environment represent potential risk and protective factors in adolescents’ everyday environments, and surveys conducted from 1981 to 2025 encompass the meaning of society development and events.

### Societal changes and significant events from 1981 to 2025

The following shifts and events illustrate adolescents’ living environment and everyday life over the last four decades in Finland and many other countries. The severe national economic recession in the 1990 s and the global recession in 2008 reduced resources for many families. The worldwide digital transition since the early 2000 s has transformed communication and increased adolescents’ exposure to both global information and misinformation. Additionally, events such as the terrorist attacks in the 2010 s, the COVID-19 pandemic, and the ongoing climate change have posed threats to young people’s futures universally. Meanwhile, family structures were diverging in modern societies, traditional families have become rarer while blended families have become more common.

Finnish new National Curriculum for Basic Education in 2014 [13] emphasized self-directed learning and the implementation of open learning environments, requiring students to develop new skills to navigate with learning effectively. Lastly, long-term changes in everyday life, such as decreased physical activity and changes in eating habits, are relevant both physical and mental wellbeing. In addition, the rising global trend of individualism [14] may contribute to a greater focus on self-interest and personal pursuits. However, if COVID-19 is not counted [15] very little is known about the association of SRH with societal changes.

### Sociodemographic factors of family and school-related factors

Poor SRH as well as mental health issues and poorer physical health have been more prevalent among adolescents from low socioeconomic families [16–18], even in the Nordic welfare states [19–21]. Not living in a two-parent family has been associated with negative trajectories in adolescents’ SRH [22, 23]. Only few studies have examined whether the association between SRH and socioeconomic factors has changed over time, considering changes in population education level and changes in family forms. Kaltiala et al. [24] found that the COVID-19 pandemic did not exacerbate socioeconomic disparities in mental health of Finnish adolescents.

On the one hand, the associations between learning, school environment and health are well-known. Low SRH is more common among low performers [17, 25], and learning difficulties are linked to academic pressures [26]. Academic pressures increase the likelihood of mental health problems [27]. In contrast, academic wellbeing and satisfaction with peer-relationships in school environment or supportive relationships with teachers are associated with higher life satisfaction [28, 29] and better SRH [25, 30, 31]. While dissatisfaction with these relationships or experiences of bullying are linked to poorer subjective health and mental health [32–34]. Less is known about whether these risk factors and their associations with SRH have changed over time.

### Gender

Gender is considered a potential modifier of the associations of SRH and its risk and protective factors, because it is a significant determinant of mental health and wellbeing across age groups worldwide [35–37]. Girls report more psychosomatic symptoms than boys [38, 39] as well as poorer mental health [40] and lower SRH [35]. Correspondingly, the decline in mental health has been more pronounced among girls [2, 26, 37, 41]. Explanations for the gender difference are diverse, such as girls and women are more health-aware and their perceived health are more affected by emotional stress compared to men and boys [6, 42].

Boys and girls also differ from each other in the risk factors for low SRH; boys’ school-performance is lower compared to girls [43], for example. Some studies have suggested that factors, such as social media use, education-related pressures, and economic recessions, have a stronger effect on girls than boys [44].

### Purpose of the study

We examine changes in low SRH among 14-year-old Finnish boys and girls from 1981 to 2025, and the association of SRH with family sociodemographic and school-related factors until 2023. Additionally, we study whether these associations changed over time and how they contributed to potential changes of low SRH.

## Methods

### Study populations

The study material consists of two datasets of 14-year-old Finns, both conducted every second year as part of Finnish national health and health promotion monitoring work. Using two datasets could extend the study from 1981 to 2025 and validate results [45].

*The Adolescent Health and Lifestyle Survey*,* AHLS.* Representative samples of 14-year-olds were drawn from the Population Registry Centre biennially between 1981 and 2019 based on birth days in June, July, and August. A self-administered questionnaire was posted in February for each survey year with two inquiries to the non-respondents. From 2009 onwards, it was possible to answer electronically. The sample sizes varied. The number of respondents ranged between 792 and 2736, in total *N* = 28 960. The response rate varied between 69 and 92% in the years 1981–1999, 45–79% in 2001–2009, and 37–60% in 2011–2019, and was lower among boys over the years. The decline in response rates in last years challenged the representativeness of the samples. Non-respondents have not been analysed systematically, but between 2015 and 2019 early and late respondents were compared according to their health behaviours, but no marked differences were observed suggesting no remarkable selection bias [46]. An example of the questionnaire is given in Supplementary 1.

*School Health Promotion Study*,* SHP.* Data consisted of national biennial classroom surveys conducted among Finnish 8th graders (14–15-year-olds), based on a total sampling from 2000 to 2025. From the latest survey 2025, only data on the proportion of low SRH was available by autumn 2025. The number of respondents ranged between 25,361 and 55,532, in total *N* = 560,446. In the 2025 survey, the number of respondents was 44, 312. The response rate varied between 63 and 84% except in 2015 (43%) due to technical problems with the digital data collection. At the national level, the study populations are representative across the study years, and non-respondents is mainly based on absence from school due to illness, travel, unauthorized absence, severe disabilities, or homeschooling or because their schools did not participate in the survey. The questionnaires and quality assessment are electronically available in School Health Promotion Study database [47, 48].

## Measures

### Outcome variable

*Self-rated health* *(SRH)* was measured with a single question: at present, would you say your health is: excellent, good, average, fairly bad, or very bad. In the analyses, the question was dichotomized. Good (excellent, good) was the reference group for low SRH (average, fairly or very bad). The dichotomization was based on previous studies and on its relevance in terms of clinical health outcomes and service use [9–11, 49, 50].

*Explanatory variables* are listed in Table 1.

**Table 1:** Explanatory variable in AHLS and SHP study

	AHLS study	SHP study
Explanatory variable		
Study year	x	x
Sociodemografic factors		
Parents’ education	x	x
Family structure	x	x
Parents’ smoking	x	x
Gender	x	x
School-related factors		
Class atmosphere	-	x
Relationships with teachers	-	x
Self-reported learning difficulties	-	x
School performance	x	-

*Survey year.* Time of the survey.

*Parents’ education* was based on students’ reports about fathers’ education. Mother’s education was not available in AHLS before 1997; the question concerned only father’s or other guardian’s education. If information about fathers’ education was missing, it was replaced by mothers’ or other guardians’ education. The variable was classified higher (0), middle (1), and lower education (2).

*Family structure* when analysed over time (from 1981 to 2023) was formed: 0 = mother and father, 1 = other family type. From 2013 onwards, we were able to consider a shared residence (children live in turn with mother and father who do not live together) using three-category variable 0 = mother and father live together, 1 = mother and father with a shared residence, 2 = one-parent family/other, such as a foster family.

*Parents’ smoking* was a proxy measure of socioeconomic circumstances. Smoking is related to family socioeconomic position [51], and it associates with adolescents’ low SRH together with other family’s sociodemographic factors [22]. The following variable was formed 1 = one or two parents smoke, 0 = parents do not smoke or have quit. In AHLS, parental smoking was no asked in 1985, 1999, 2005–2007, 2017–2019.

*Gender* was binary (boy, girl).

*School-related variables in SHP study.* Three school-related variables were construed from the corresponding survey questions, and an explorative factor analysis was used to confirm that they represent distinct aspects of the school environment.

- *Relationships with teachers* were asked with the questions: teachers encourage me to express my opinions in class, teachers are interested in how I am doing, teachers treat us students fairly. The options were fully agreed, agreed, disagreed, fully disagreed. Each question was scored 1 (fully disagreed, disagreed) or 0 (fully agreed, agreed) and a sum variable was composed. The variable range was 0 − 3.
- *Class atmosphere* covered two items: it’s peaceful to work in my class, the students in my class like being together. The options (fully agree, agree, disagree, fully disagree) were scored into two categories 1 (fully agree, agree) or 0 (disagree, fully disagree). A sum variable was composed. The range was 0 − 2.
- *Self-reported learning difficulties* were measured with the question: Are you experiencing difficulties in the following things at school: teaching in class, doing homework or other similar tasks, preparing for exams, performing tasks that require writing, performing tasks that require reading. The following scoring system was used: 1 = yes (very much or quite a lot), 0 = no (not at all or little). The scores were summed to create a sum variable (the range 0 − 5).

*School-related variables in AHLS study.* One school-related question could be analysed across the years.

- *School performance* was the respondents’ report of his/her performance compared to the class average with the options: much better, slightly better, about class average, slightly poorer, much poorer. A classified variable was used in the analyses, lower (slightly or much poorer), average, better (much or slightly better).

### Statistical methods

Gender-stratified analyses were performed separately for SHP and AHLS datasets in three steps.

Step 1) Changes in low SRH over time was examined using relative proportions.

Step 2) Logistic regression modelling was used to analyse Odds Ratios (ORs) of low SRH by each explanatory variables and exploring changes in these associations over time. First, each explanatory variable was added separately into the logistic regression model adjusted for the survey year (Models 1). Next all explanatory variables were added to the model (Models 2). In these models, if the variable had more than 5% missing values [52], the missing data were considered as a separate category, but their results are not presented.

Changes in the associations between low SRH and explanatory variables (from 1981 to 2023) were analysed by adding the interaction term of the explanatory variable and the survey year into Models 1. To streamline the results, the following explanatory variables were dichotomized; high educated parents/others, high school performers/others, and for school-related sum variables no (scores = 0)/yes (scores = 1 or more). Then, yearly ORs for low SRH were calculated by the explanatory variable and adjusted for parents’ education. This was interpreted as a potential confounder when analysing school-related and other family-related variables. In addition to ORs, average marginal effects (AMEs) were reported for each explanatory variable by study year. AMEs provide comparable effect sizes by quantifying differences in predicted probability, and these can be interpreted directly as percentage‑point changes in the associations.

Step 3) Finally, the Fairlie method, an extension of Blinder-Oaxaca decomposition method [26, 53], was used to investigate how much of the observed change in low SRH accounted for the changes in the explanatory variables over time. The Fairlie method compares two groups by decomposing a multivariate logistic regression model into components and returns a table showing how much of the absolute difference between two groups can be explained by the difference of each explanatory variable in the model. The positive coefficient of the explanatory variable implies that differences in its distribution between the groups widen the observed gap in the outcome variable. The negative coefficient implies that the difference in the distribution of that variable between the groups reduces the difference. Here, comparable groups were the survey years, whose selection was based on the observed change. The explanatory variables were included into the model as dichotomous.

The group effect of the hierarchical school data of SHP was analysed by calculating intraclass correlations (ICC). ICC of low SRH between schools was very low (0.3% among boys, 0.5% among girls) which is why the school level effect was omitted from the analyses [54, 55].

In AHLS, *p*-values < 0.05 were considered statistically significant while in SHP, the threshold value was *p* <.01 due to the large sample size. Data were analysed using SPSS (V.28.0) and Stata (V.18.0).

## Results

### Changes in low SRH over time

**Fig. 1:**
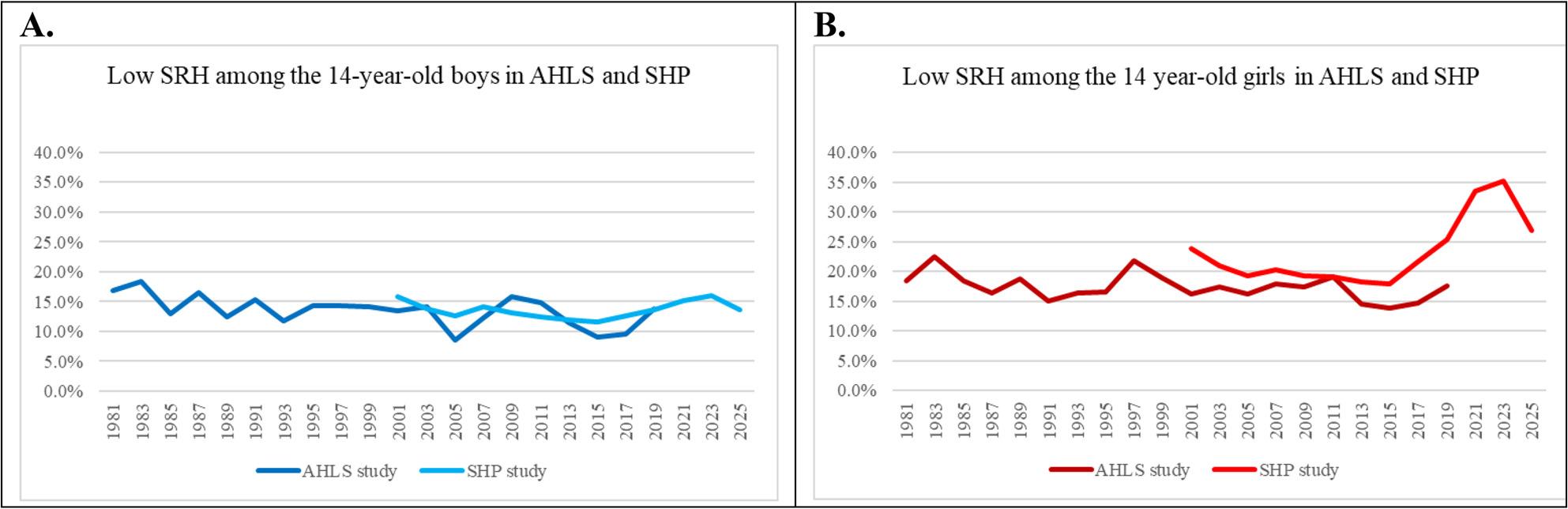
Low self-rated health (%) among 14-year-olds by gender, in AHLS and SHP. **A**) Boys. **B**) Girls

The proportions of those with low SRH from 1981 to 2025 are presented in Fig. 1. The notable change was the increase in girls’ low SRH from 2015 to 2023, followed by a decrease between 2023 and 2025. No corresponding change was observed among boys. The proportions for each category of self-rated health over the years are provided in Supplement 2.

### Variation in low SRH based on sociodemographic and school-related factors

Tables 2 and 3 show that low SRH was more prevalent among adolescents whose parents had lower education levels or smoked, who did not live in a two-parent family, and whose school performance was average or poor. Additionally, low SRH was more prevalent if class atmosphere was not supportive, relationships with teachers were not trusted or encouraging, and if the respondent had learning difficulties.

**Table 2:** ORs for low self-rated health according to sociodemographic and school-related factors in AHLS

Explanatory variable	Boys (*N* = 14 225)						Girls (*N* = 16 547)					
	Model 1^a^			Model 2^b^			Model 1^a^			Model 2^b^		
	OR	95% CI		OR	95% CI		OR	95% CI		OR	95% CI	
Parents’ education												
High	1			1			1			1		
Middle	1.05	0.90	1.22	0.89	0.76	1.04	**1.42**	1.24	1.62	1.15	1.00	1.32
Low	**1.30**	1.09	1.57	1.00	0.82	1.21	**1.81**	1.54	2.11	**1.29**	1.09	1.52
Family structure												
Mother and father	1			1			1			1		
Other	**1.41**	1.25	1.58	**1.26**	1.11	1.44	**1.47**	1.34	1.62	**1.21**	1.09	1.34
Parents’ smoking^1^												
No	1			1			1			1		
Yes	**1.36**	1.20	1.54	**1.15**	1.01	1.32	**1.53**	1.38	1.69	**1.25**	1.12	1.39
Self-reported school performance (compared to classmates)												
Better	1			1			1			1		
Average	**1.32**	1.16	1.49	**1.28**	1.13	1.45	**1.96**	1.78	2.15	**1.85**	1.68	2.04
Lower	**2.47**	2.17	2.81	**2.27**	1.98	2.61	**3.80**	3.36	4.29	**3.41**	3.00	3.88

Statistically significant marked bold

^a^Model for each explaining variable, adjusted for study year

^b^Model adjusted for all explaining variables and study year

^1^Parents’ smoking was not asked in 1981, 1983, 1989, 2009 and 2011, but these years were included in the Model 2 by adding missing values as their own category (not shown)

**Table 3:** ORs for low self-rated health according to socio-demographic and school-related factors in SHP study

Explanatory variable	Boys (*N* = 280 186)						Girls (*N* = 279 784)					
	Model 1^a^			Model 2^b^			Model 1^a^			Model 2^b^		
	OR	99% CI		OR	99%CI		OR	99% CI		OR	99%CI	
Parents’ education^1^												
High	1			1			1			1		
Middle	**1.14**	1.10	1.18	**1.16**	1.10	1.23	**1.27**	1.23	1.30	**1.24**	1.18	1.29
Low	**1.51**	1.44	1.59	1.02	0.98	1.06	**1.68**	1.62	1.75	**1.06**	1.03	1.10
Family structure^2^												
Mother and father	1			1			1			1		
Other	**1.70**	1.64	1.75	**1.34**	1.30	1.39	**1.68**	1.63	1.72	**1.29**	1.26	1.33
Family structure^3^												
Mother and father live together	1			1			1			1		
A shared residence	**1.23**	1.14	1.32	**1.09**	1.00	1.18	**1.26**	1.19	1.33	**1.07**	1.00	1.15
One-parent or other arrangements	**1.95**	1.85	2.06	**1.49**	1.40	1.58	**1.81**	1.74	1.88	**1.32**	1.26	1.38
Parents’ smoking												
No	1			1			1			1		
Yes	**1.60**	1.55	1.65	**1.30**	1.26	1.35	**1.70**	1.66	1.74	**1.29**	1.26	1.33
Class atmosphere												
Supportive	1			1			1			1		
Some problems with peace or being together	**1.65**	1.60	1.70	**1.38**	1.33	1.43	**1.48**	1.44	1.52	**1.24**	1.21	1.28
Problems with peace and being together	**2.69**	2.58	2.80	**1.99**	1.90	2.08	**2.13**	2.06	2.19	**1.62**	1.57	1.68
Relationship with teachers												
Trusted and encouraging	1			1			1			1		
Average	**1.35**	1.30	1.41	**1.16**	1.11	1.21	**1.55**	1.50	1.61	**1.27**	1.23	1.32
Some good elements	**1.86**	1.78	1.94	**1.39**	1.33	1.46	**2.22**	2.15	2.30	**1.54**	1.48	1.60
Poor	**2.77**	2.65	2.89	**1.72**	1.63	1.80	**3.61**	3.48	3.75	**2.02**	1.94	2.11
Number of self-reported learning difficulties												
none	1			1			1			1		
1	**1.81**	1.74	1.89	**1.65**	1.57	1.73	**2.24**	2.17	2.32	**1.97**	1.90	2.04
2	**2.44**	2.34	2.55	**2.09**	1.99	2.20	**3.33**	3.21	3.45	**2.76**	2.66	2.87
3	**3.08**	2.93	3.23	**2.52**	2.39	2.66	**4.65**	4.47	4.84	**3.66**	3.51	3.82
4	**3.87**	3.66	4.08	**3.03**	2.85	3.22	**5.63**	5.37	5.90	**4.20**	3.99	4.42
5	**4.65**	4.40	4.91	**3.39**	3.19	3.61	**7.35**	6.98	7.74	**5.25**	4.96	5.56

Statistically significant marked bold

^a^Model for each explaining variable, adjusted for study year

^b^The model adjusted for all explaining variables and study year

^1^Missing values (9.3% among boys and 7.4% among girls) were included in the model as their own category (not shown)

^2^Family structure from 2000 to 2023

^3^Family structure from 2013 to 2023

The associations of low SRH with average or low school performance, self-reported learning difficulties, poorer relationship with teachers, and parental lower education were stronger among girls compared to boys. Conversely, a poorer classroom atmosphere was more strongly associated with low SRH among boys. In the multivariable models (Tables 2 and 3), the associations between the explanatory variables and low SRH generally persisted, only parents’ lower education lost statistical significance among boys in AHLS. Gender differences in school-related factors did not change. In AHLS, the multivariate model explained 3.9% of the variation in low SRH among girls and 2.3% among boys. The corresponding rates in SPH were 10.5% and 6.6%.

### Changes in the associations of low SRH with sociodemographic and school-related factors from 1981 to 2023

**Fig. 2:**
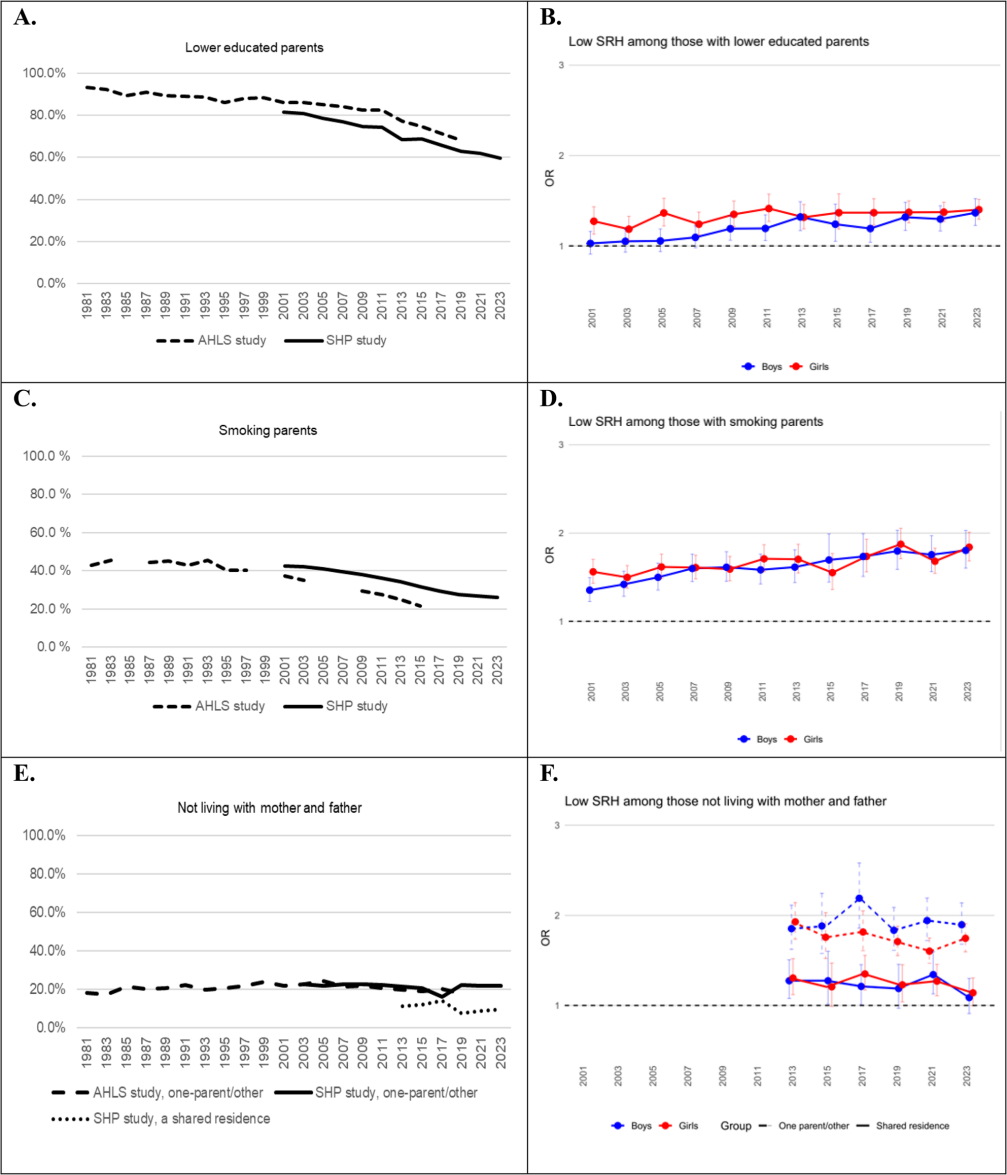
Prevalence of sociodemographic family factors and ORs for low self-rated health by these factors in 1981–2023

The percentages of lower educated parents and smoking parents steadily decreased from 1981 to 2023, Fig. 2, A, C. Between 2000 and 2023, the association of parents’ lower education with low SRH slightly strengthened among boys (OR 1.01 per year, p = < 0.001) but diminished among girls (OR 0.98 per year, p = < 0.001), Fig. 2, B. Girls from lower educated families reported less often low SRH than was expected based on the main effects of the study year and parents’ education. In the late 2000 s, boys had higher ORs for low SRH in smoking families OR 1.02 per year, p = < 0.001), compared to the early 2000 s, while among girls the association stayed stable over time, Fig. 2, D.

The percentage of children not living in a two-parent family persisted over time, Fig. 2, E, and the association of low SRH between the two-parent family and a one-parent/other family type did not change over time. In 2013–2023, around 10% of adolescents reported a shared residence (living in turn with mother and father), Fig. 2, E. When the shared residence group was compared to traditional two-parent families, the OR for low SRH did not change from 2013 to 2023 in shared residence (OR 0.982, *p*=.250 among boys, OR 0.977, *p*=.076 among girls), Fig. 2, F. The association of low SRH between traditional family and one-parent/other family type decreased statistically significantly over the years among girls living with one-parent/other family (OR 0.97 per year, *p*=.006), but stayed stable among boys (OR 1.01, *p*=.637), Fig. 2, F from 2013 to 2023.

**Fig. 3:**
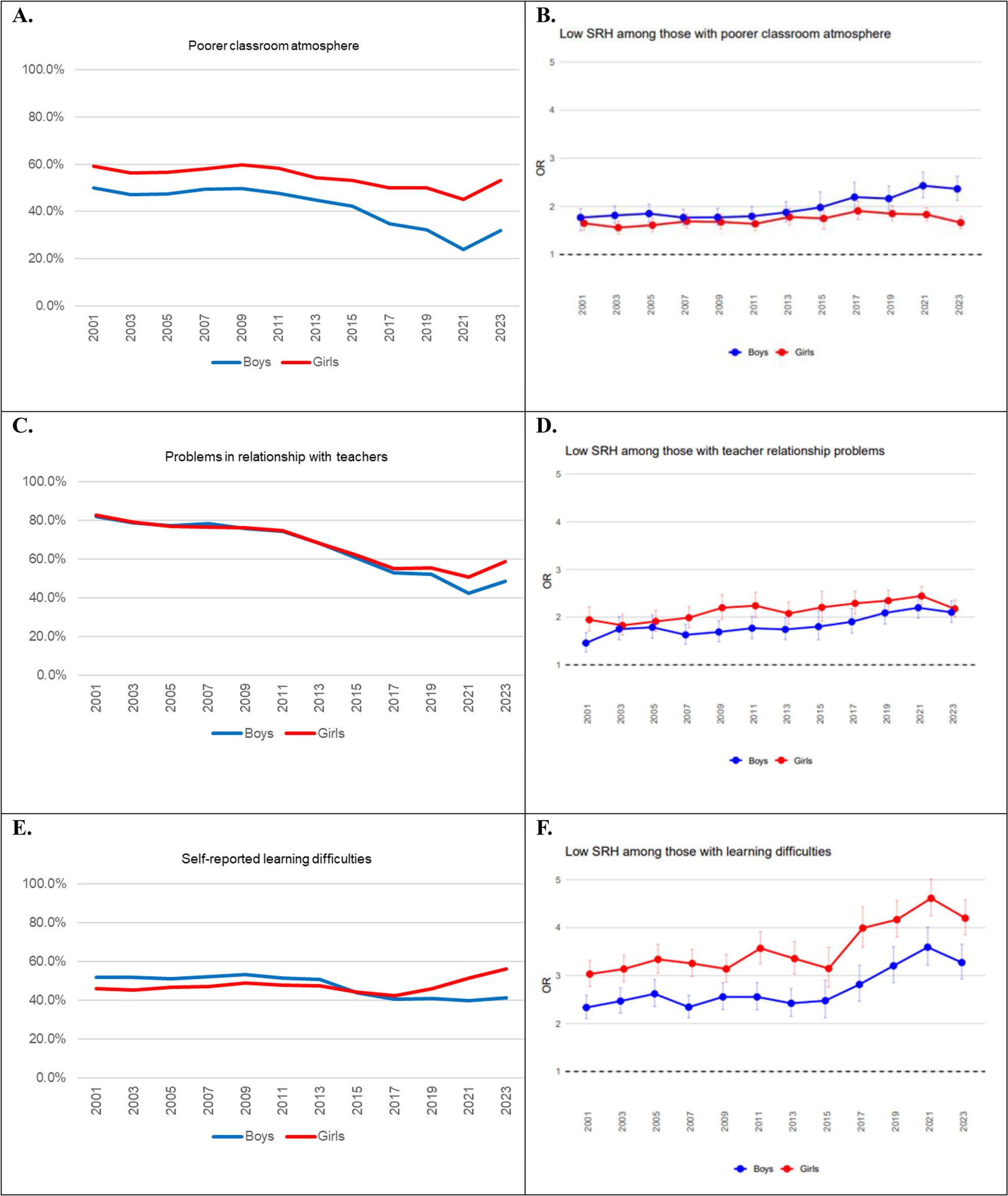
Prevalence of school-related factors and ORs for low self-rated health by these factors in 2000–2023

From 1981 to 2019, the proportion reporting poorer school performance compared with classmates decreased among boys and remained steady among girls. Its association with low SRH remained stable (OR 1.01, *p*=.303 among boys, OR 1.00, *p*=.823 among girls, not shown in Fig. 3). The proportion of children reporting poorer class atmosphere and problems in relationships with teachers decreased from 2000 until 2021 but turned to an increase from 2021, Fig. 3, A, C. During the study period, the odds for low SRH increased among boys with poorer class atmosphere (OR 1.02 per year, p = < 0.001) or problems in their relationship with teachers (OR 1.02 per year, p = < 0.001) compared to those with better atmosphere or better relationship with teachers, Fig. 3, B, D. Among girls, the associations between low SRH and poorer classroom atmosphere (OR 1.00, *p* =.212) and problems in relationship with teachers (OR 0.99, *p* =.075) did not change over time.

The proportion of adolescents reporting learning difficulties stayed stable until 2017, after which it increased among girls but decreased among boys, Fig. 3, E. The OR for low SRH increased between 2015 and 2023 among those with learning difficulties compared to those without (OR 1.03 per year, p=.<0.001 among boys; OR 1.04 per year, p = < 0.001 among girls), Fig. 3, F.

The trendlines in yearly ORs or goodness of fit values (AIC and BIC) in the above school models did not change substantially, when other school-related factors were included in the models.

The marginal effects on low SRH according to all explanatory variables across the study years are available in Supplements 3–4.

### Changes in sociodemographic and school-related risk factors and their contribution to changes in low SRH

The proportion of low SRH among girls increased from 17.9% in 2015 to 35.2% in 2023. The decomposition analysis showed that changes in sociodemographic and school-related factors were associated with the change in girls’ low SRH. Together these changes accounted for 8.8% of the change in low SRH (Table 4). Increased learning difficulties had the greatest contribution to the increased OR for low SRH among girls.

**Table 4:** Contribution of changes in family sociodemographic and school-related risk factors to the increase in girls’ low self-rated health between 2015 and 2023, Decomposition analysis among girls (n= 29 769)

Expalanatory variable	coefficents^1^	*p*
Parents’ lower education	0.0010	0.001
A shared residence	0.0000	0.734
Living with a single parent/other arrangements	−0.0021	< 0.001
Parents’ smoking	0.0004	< 0.001
Poorer class atmosphere	−0.0016	< 0.001
Problems in relationships with teachers	0.0009	< 0.001
Self-reported learning difficulties	−0.0136	< 0.001
All together	−0.015	
Total change among girls between 2015 and 2023	−0.171	100%
Change explained by the changes in variables in the model	−0.015	8.8%

^1^For the change in prevalence from 2023 to 2015

For the boys, no significant changes in low SRH were observed over time and the decomposition analysis was not applied.

## Discussion

The main finding was that the proportion of adolescents reporting low SRH remained steady until 2015, after which an increase emerged among girls, peaking in 2023. The most notable strengthening association over time was observed between low SRH and learning difficulties among both genders. All sociodemographic and school factors were associated with low SRH across the surveys. Approximately 9% of the increase in low SRH among girls was attributable to changes in these variables and learning difficulties were the only factor that contributed to the decline in girls’ SRH.

### Trends in SRH

Only a few other studies on SRH with long-term trends have been published so far. The longest trend from a Norwegian study showed little change in poor SRH from 1995 to 1997 to 2006 − 2008 and an increase to 2017–2019 among girls and older adolescents [50]. Other studies describing trends are country-based on HBSC study between 2002 and 2022. These have shown rather stable trends in low SRH with some variation until 2014/2017 [49, 56, 57], an increase in excellent SRH between 2002 and 2006 [35, 58], and a rise in low SRH from 2017 to 2022 among girls and older adolescents [49]. Overall trend appears like our findings: an increase in low SRH during the later study years, particularly among girls.

### Societal changes and crises

Our study period from 1981 to 2025 was marked by several societal changes and national and international crises. However, we observed changes only after 2015 in the otherwise stable proportion of adolescents experiencing low SRH. Consequently, the association between low SRH and societal changes was not straightforward. Earlier studies have suggested that children’s subjective wellbeing is less strongly associated with macro-level societal factors and more with cultural context and satisfaction within their immediate environments – specifically family, peers, and the school environment [29, 59, 60].

The rise of low SRH began from 2015 onwards, peaking during the COVID-19 pandemic (2020–2022) and levelling off in 2025. The peak between 2020 and 2022 refers to the specific impact of the pandemic. Compared to other crises, the serious restrictions on physical and social contacts, school closures, threats of illness (to the adolescent or their parents), and adverse effects on family economy penetrated more deeply into adolescents’ lives during the COVID-19 pandemic. The pandemic, however, cannot explain why the declining SRH was observed only among girls.

The decline in girls SRH was observed even before the pandemic. The onset of this decline in 2015 coincides with the increasing use of the internet and social media. During 2020–2021, 10–14-year-old Finns averaged nearly four hours of screen time daily [61]. While the pandemic’s mitigation measures restricted physical interactions with peers, they concurrently facilitated a shift toward digital communication. Increasing evidence shows that while social media can offer opportunities, it also poses risks to adolescents’ wellbeing and mental health, particularly among girls [62].

### Somatic health and physical functioning

While worsening somatic health among adolescents could potentially explain changes in their SRH, too, current population-level evidence does not support this. Between 1981 and 2015, prevalence of low SRH remained stable even though e.g., overweight, asthma, allergies, and diabetes increased among Finnish children [63–66]. On the other hand, data from Finnish 14-year-old girls from 2019 to 2022 showed a rise in the proportion of those whose physical functioning was so low that it could jeopardize their health and wellbeing [67]. This increase coincided with the pandemic years and did not persist in subsequent years, and it was not observed among boys. We cannot exclude the possibility that this decline has contributed to the decrease in girls’ SRH.

### Sociodemographic factors

Regarding the associations between sociodemographic factors and low SRH, our results are in line with previous studies, which have shown that children from lower socio-economic background more frequently report low SRH [17, 20, 22, 25, 68]. Richter et al. [25] emphasized the significance of material factors in explaining the association of family affluence with SRH, although it has been shown that low socioeconomic circumstances are associated with low SRH, the association has varied depending on the indicators and the country [17]. Unlike earlier studies, we had data on shared residence, suggesting that children who live with the parents in turn (usually weekly) are less likely to have low SRH compared to one-parent families or other arrangements. However, low SRH was still more common than in families where the parents lived together.

In a previous Finnish study, socioeconomic differences in low SRH of adolescents remained relatively stable over time [69]. In our study, the association of parental smoking and parental lower education with low SRH slightly increased among boys over the years while among girls, the association of low SRH with parental lower education and family type slightly decreased. Some gender-specific underlying mechanisms that affect SRH might explain this, but we cannot exclude chance variations in children’s answers over the years. Other studies have reported changing SES patterns, too. In many countries life satisfaction has declined more among girls and higher SES groups in recent years [70]. It is known that life satisfaction and SRH are correlated [56].

### School-related factors

Our results revealed several changes in school-related factors, both in prevalence and in the associations with low SRH. The improvement in the class atmosphere and relationships with teachers from 2001 until 2021 indicates favourable trends in Finnish schools. This can be attributed, at least in part, to gradual changes outlined in the National Core Curriculum, which emphasized for more student-centred learning methods and pedagogy [13, 71]. At the individual level, our results showed a strong relationship between SRH and both class atmosphere and teacher relations. Earlier studies have reported similar findings; students who experience a poorer classroom atmosphere, or weaker relationships with teachers are more likely to report low SRH [25, 31, 33, 34]. Based on these favourable trends, we anticipated improvements in SRH over time. However, this was not observed, and the likelihood of low SRH slightly increased among those boys who reported a poor class atmosphere or problems in teacher relationships. It is possible that boys with low SRH represent a more vulnerable group over time. As negative school-experiences decline in the general school population, the association with other vulnerabilities, such as low SRH, appears to strengthen. A similar strengthening was observed in the association between lower family sociodemographic factors and low SRH among boys, but this was not the case among girls.

We also observed that self-reported learning difficulties were strongly related to low SRH, and this association has strengthened since 2017, after the new National Core Curriculum was implemented. During this period, the experiences of learning difficulties increased among girls, but slightly decreased among boys. Earlier studies have demonstrated a relationship between SRH and school performance [17, 25], as well as the strengthened association between lower school marks and school related stress [72]. School-related stress has been suggested to contribute to adolescents’ emotional problems [12], but none of the studies have specifically addressed learning difficulties or the strengthening of the relationship between SRH and school performance. International PISA studies have shown a decline in the academic achievement of Finnish schoolchildren for years, with the most significant decline occurring among the low-performing group since 2012, where boys are overrepresented [43]. Given that SRH is known to correlate with mental health [73], our results raise concerns about the intertwined association between the trends of increasing mental health problems and declining school performance.

Overall, it appears that a subset of children with negative school experiences is increasingly distinct from the broader school population, a differentiation that has widened during the recent years. These students may also belong to low-performing group. Those with the poorest knowledge and skills often share characteristics such as having parents with lower levels of education and lacking traditional higher socioeconomic status indicators in their homes [74]. However, our data is too limited to comprehensively illuminate these changes over time or to assess the influence of other factors, such as the education system reforms and the digitalization of learning. Nonetheless, an increase in the number of children with poorer school experiences and low SRH may lead to polarization, contributing to marginalization within this group and health inequalities in later life, and an increasing burden on the health sector.

### Mental health

The explanations for the increasing gender difference in SRH are likely diverse, but our finding aligns with earlier studies of adolescents’ mental health where gender gap has widened in recent years [2, 26, 37, 41]. Consequently, the increase in girls’ low SRH refers genders differences in mental burden, or at least how it is reported. Previous studies suggest that particularly social media platforms and school pressures have contributed gender difference in mental health issues in recent years [44].

The change in girls’ low SRH coincides with a new operational logic of social media platforms – personalized and algorithm-optimized content. Body image concerns and the social pressure to conform to beauty standards amplified by social media have been suggested to negatively impact mental health, particularly among girls [62]. Further, previous research indicates a strong association between body image concerns and self-esteem among girls [75]. When self-esteem plays a significant role in adolescents’ SRH, too [7, 30, 75], self-esteem is a potential mechanism linking SRH to social media influences.

The rise in girls’ low SRH also falls within a period during which adolescents spent more time than ever before in front of screens. During to the COVID-19 pandemic, a variation in the type of screen time was observed between genders among 10–14-year-olds: girls at this age group spent more than three times more time on social media platforms compared to boys [61]. On the other hand, excessive internet use slightly declined among Finnish adolescents in 2023–2025 [48] when the decline in girls’ SRH levelled off.

School pressure has increased more over time among girls than among boys [76]. We did not have a measure of school pressure in our study, but we showed an increase in the proportion of girls reporting learning difficulties, which may also reflect school pressures [26]. Other studies have shown that the growing gender difference in psychosomatic symptoms and mental health can be attributed to gender differences in school pressures at least partly [37, 39]. It is possible that gender difference in low SRH align the same pattern. Furthermore, 2014 basic education curriculum reform which emphasized students’ self-directed learning, peer and self-assessment may have gradually boosted girls’ more critical self-ratings [13]. When students are directed continuously to do peer and self-assessment, some of them, especially girls, may fall short from both external and their own expectations leading gradually to lowered wellbeing.

In addition, a few cross-country studies have suggested that macrolevel factors in society may influence the gender difference in mental health symptoms among adolescents. In these studies, gender difference has widened more in those countries, which had a higher gender-equal index [37, 40]. These unexpected findings emphasize to investigate also the macrolevel factors, such as socio-cultural contributors, behind the gender differences.

### Strengths and limitations

To our knowledge, this study has the longest time series of adolescents’ low SRH published so far. The sampling frames differed between SHP and AHLS. SHP was based on classroom surveys covering all 8th grades (14-year-olds) in the country, while AHLS was a mailed survey to home addresses aimed to nationally representative samples of 14-year-olds. The two surveys overlapped eighteen years; during these years the trends followed the similar pattern, which strengthens the interpretation of valid findings. The representativeness of AHLS in the later survey years was challenged by the declining response rate and in SHP in 2014 survey, where technical problems disturbed the data collection. We cannot rule out selection bias, particularly in the latter years of AHLS.

Our sample sizes were sufficiently large to detect systematic changes in the associations over time. However, in large samples, trivial differences in the associations can achieve statistical significance without practical relevance. Here, our focus was on identifying systematic changes over time, but still effect sizes remained weak in some cases, such as the changes in the associations between low SRH and sociodemographic factors. On the one hand, by focusing only on systematic changes, more complex patterns may have remained undetected. Future research should also examine non‑linear trends and thresholds to better understand the observed dynamics in SRH over time.

The number of explaining variables was limited due to changes in the questions over time and due to differences in the available questions between the data sets. This required compromises in the selection of the variables and in the number of categories in some variable, when creating comparable measures in both data sets over time. Even if the formulation of the question stays similar, the socio-cultural changes over time may change the answering tendency. All our variables were self-reported, which has some limitations, too. We did not have objective measures of school performance and learning difficulties. Children do not necessarily know their parents’ education level; in school surveys they cannot check this but in mailed surveys they may ask their parents. Large sample sizes have minimized this individual-level random variation. Our study examined associations between low SRH and explanatory variables and direct causal links were not our assumption.

## Conclusions

The study illuminates the association between social determinants and adolescents’ SRH over time. Finnish adolescents’ low SRH stayed steady from 1981 until 2015, despite societal changes, improvements in family sociodemographic indicators and positive changes in school environment. The declining SRH among girls after 2015 aligns with finding of lowering mental health during the past years. The steeper decline in SRH between 2021 and 2023 and the levelling off in the subsequent years can be attributed specifically to the pandemic related factors. There is no simple explanation to the gender gap in SRH and why the increase in the proportion of adolescents reporting low SRH was observed only among girls. However, school-related stress and the increasing use of the internet and social media appear to be strong candidates for explaining the changes observed among girls after 2015. A practically important finding for schools was the strong association between low SRH and self-reported learning difficulties among both genders, and particularly that this association strengthened over time. This was not expected because of improvements in perceived school social environment. Further research is necessary to reveal factors that influence adolescents’ wellbeing and learning in contemporary society.

## Supplementary Information

Supplementary Material 1

Supplementary Material 2

Supplementary Material 3

Supplementary Material 4

## Data Availability

The datasets in this article are not publicly available. The original dataset of School Health Promotion Study is available upon reasonable request info(at)findata.fi, and the dataset of Adolescents Health and Lifestyle Survey by contacting Jaana M Kinnunen (jaana.kinnunen(at)tuni.fi).
