# Supplementary material for: Changes in self-rated health and its association with social determinants – repeated cross-sectional surveys among Finnish adolescents from 1981 to 2025
Source: BMC Public Health. 2026 Mar 18;26:1375. doi: 10.1186/s12889-026-27022-y (PMC13112845; doi:10.1186/s12889-026-27022-y)
Supplement: Supplementary file 1 — Supplementary Material 1 [file 12889_2026_27022_MOESM1_ESM.pdf]

AN EXAMPLE OF A SURVEY QUESTIONNAIRE, YEAR 1999

UNIVERSITY OF TAMPERE
Tampere School of Public Health
POB 607 (Medisiinarinkatu 3)
33101 TAMPERE

# ADOLESCENT HEALTH & LIFESTYLE SURVEY

Dear Adolescent!

We are making a survey on the health and welfare of young people. You have been chosen as a representative of Finnish adolescents.

Please read the questions carefully and answer those relating to you. The questionnaire was mailed to 12-18 year olds. The questions directed to the higher age groups may not all be relevant to the lower age groups at present.

We hope that you can reply within a week. You may return the form in the enclosed envelope postage pre-paid. The information contained in the questionnaire will be stored with an optical reader, therefore please do not fold the form when inserting it in the envelope.

All data on the questionnaire will be dealt with confidence. The forms are marked with reference numbers in order to avoid remailing to those who already responded.

If your parents wish to see the questionnaire we hope they will do so before you answer. In order to ensure the reliability of the survey it is highly important that everyone answers the questions independently. Your address information was obtained from the population information systems (Population Register Center, P.O.Box 7, 00521 Helsinki). If you have any questions concerning the survey please call our research secretary Mrs Margareta Ekman, Tampere, phone 03-215 7926.

February 1999

Arja Rimpelä
Professor
Tampere School of Public Health
University of Tampere

## FILLING INSTRUCTIONS

Read first the whole question. Answer the question by ticking the box at the correct or most suitable alternative. If no alternatives are given write your answer on the line provided. Use soft pencil, ballpoint or fountain-pen. If you make a mistake DO NOT ERASE YOUR ANSWER, instead blacken the entire box with the wrong answer and then tick the correct alternative.
Like this:

☐ wrong answer ☐ correct answer

Example: If you eat dinner every day, you would answer the following question like this:

**How often do you eat dinner (a warm meal in the afternoon or in the evening)?**

- ☐ every day
- ☐ about 3-4 times a week
- ☐ about once a week or less often

Some questions are followed by a note that you may proceed directly to a later question as marked with a number. In this case you need not answer any questions remaining in between.

---

1 **Sex**

- ☐ girl
- ☐ boy

2 **Date of birth____ /____ 19____**

3 **Height ________cm**

4 **Weight _________kg**

5 **Where kind of place do you live in?**

- ☐ CITY, in the centre or in a suburb
- ☐ CITY, outside the centre and the suburbs
- ☐ COUNTRYSIDE, in the village centre
- ☐ COUNTRYSIDE, outside the village centre

6 **What is your father's or other guardian's main profession, work or occupation?**

PLEASE GIVE YOUR DESCRIPTION AS PRECISELY AS POSSIBLE.

(E.g. machine fitter, primaryary school teacher, editor.) Do not use general names (e.g. worker, teacher), degrees and titles (e.g. Bachelor of Bus., M.A. municipal counselor). If retired, write former occupation (e.g. retired, sheet worker).

____________________________

7 **What kind of work is your father or other guardian doing for most of the year? If retired or unemployed at present, give former work.**

- ☐ planning, administrative, teaching, research, or other corresponding work
- ☐ supervisory work in industry, building, forestry, mining etc.
- ☐ manual work in industry, building, forestry, mining etc.
- ☐ work in office, shop, restaurant or other corresponding work
- ☐ farming or other farm work
- ☐ other work, which? ____________

8 **Who belong to your family?**

- ☐ mother and father
- ☐ mother and stepfather
- ☐ father and stepmother
- ☐ mother only
- ☐ father only
- ☐ husband or wife (married or common law)
- ☐ other guardian, who? __________

9 **Do you have a steady relationship at the moment?**

- ☐ yes
- ☐ no

**10 What is your father's education?**

- ☐ primary or primary and secondary school only
- ☐ primary or primary and secondary school and vocational school (trade school, courses etc.)
- ☐ old comprehensive school (and trade schools etc.)
- ☐ matriculation examination (and trade schools etc.)
- ☐ college or university graduate (M.A., B.Sc.Eng., etc.)

**11 What is your mother's education?**

- ☐ primary or primary and secondary school only
- ☐ primary or primary and secondary school and vocational school (trade school, courses etc.)
- ☐ old comprehensive school (and trade schools etc.)
- ☐ matriculation examination (and trade school etc.)
- ☐ college or university degree (M.A., B.Sc.Eng., etc.)

## SCHOOL AND WORK

**12 What are your plans for future education?**

- ☐ will finish upper secondary school, then apply to university or college
- ☐ will finish upper secondary school, but probably not apply to university or college
- ☐ will complete a vocational school
- ☐ will not continue my studies
- ☐ don't know

**13 Are you attending a school or studying at present?**

- ☐ I do not go to school and I am not a student (proceed to Question 18)
- ☐ I go to school or study full-time
- ☐ I work and I also go to school or study

**14 What kind of school are you attending?**

- ☐ primary/secondary or corresponding school
- ☐ upper secondary school
- ☐ university, college, higher trade school
- ☐ vocational institution
- ☐ course for unemployed, trade course etc.
- ☐ other, which ?____________________

**15 What kind of grades did you receive last? If you compare them with the average level of your class or course, were they**

- ☐ much better
- ☐ slightly better
- ☐ about class average
- ☐ slightly poorer
- ☐ much poorer

**16 Is smoking permitted in your school?**

- ☐ not permitted at all
- ☐ permitted in restricted areas
- ☐ permitted without restrictions

**17 How carefully does your school control the smoking restrictions?**

- ☐ very carefully
- ☐ to some extent
- ☐ seldom

## HEALTH

**18 How do you feel about your health? Is it at present**

- ☐ excellent
- ☐ quite good
- ☐ average
- ☐ rather bad
- ☐ very bad

**19 Do you have a chronic disease, injury or disability that restricts your daily activities?**

- ☐ no
- ☐ yes

**20 Do you take regularly or nearly regularly any medicine prescribed by a doctor?**

- ☐ no
- ☐ yes, which?____________________

**21 During the past SIX MONTHS, have you had any of the following symptoms and how frequently? Tick the alternative that suits you best on each line.**

| | Seldom or not at all | About once a month | About once a week | Nearly every day |
|---|---|---|---|---|
| Stomach aches | ☐ | ☐ | ☐ | ☐ |
| Tension or nervousness | ☐ | ☐ | ☐ | ☐ |
| Irritability or outbursts of anger | ☐ | ☐ | ☐ | ☐ |
| Trouble falling asleep or waking up in the middle of the night | ☐ | ☐ | ☐ | ☐ |
| Headache | ☐ | ☐ | ☐ | ☐ |
| Hands shaking | ☐ | ☐ | ☐ | ☐ |
| Feeling tired or weak | ☐ | ☐ | ☐ | ☐ |
| Feeling dizzy | ☐ | ☐ | ☐ | ☐ |

**22 During the past SIX MONTHS, have you had lower back pains?**

- ☐ seldom or not at all
- ☐ about once a month
- ☐ about once a week
- ☐ nearly every day

**23 During the past SIX MONTHS, have you had neck or shoulder pains?**

- ☐ seldom or not at all
- ☐ about once a month
- ☐ about once a week
- ☐ nearly every day

**24 During the past MONTH, have you often felt blue, depressed or hopeless?**

- ☐ yes
- ☐ no

**25 During the past MONTH, have you often felt that nothing interests you or brings you pleasure?**

- ☐ yes
- ☐ no

**26 When have you last visited a doctor (other than dentist) on account of injury or illness?**

- ☐ this year (1999)
- ☐ in 1998
- ☐ in 1997 (proceed to Question 28)

**27 How many times have you visited a doctor last year (1998) and this year altogether on account of injury or illness?**

- ☐ once
- ☐ twice
- ☐ 3-4 times
- ☐ 5-10 times
- ☐ more than 10 times

**28 Have you used any mental health services last year (1998) or this year (e.g. psychiatric clinics for children or youth, family counseling, psychologist or psychiatrist)? How many times altogether?**

- ☐ never
- ☐ once
- ☐ twice
- ☐ 3-4 times
- ☐ 5-10 times
- ☐ more than 10 times

**29** What do you think of your weight? Do you think you

- ☐ need to lose a lot of weight
- ☐ need tolose some weight
- ☐ need not lose or gain any weight
- ☐ need to put on some weight
- ☐ need to put on a lot of weight

**30** How often do you brush your teeth?

- ☐ never
- ☐ about once a week or less
- ☐ two or three times a week
- ☐ about 4-5 times a week
- ☐ about once a day
- ☐ several times a day

## ACCIDENTS

**31 During the past MONTH, have you had an accident that needed to be taken care of by doctor or nurse?**

- ☐ no (proceed to Question 36)
- ☐ yes, how many times?
  - ☐ once
  - ☐ twice
  - ☐ 3 times or more

**When you answer Questions 32-35, think of your MOST SERIOUS accident during the past month.**

**32 Where did this accident occur?**

- ☐ at home
- ☐ at school
- ☐ on the way to school
- ☐ elsewhere in traffic
- ☐ in connection with sports activities (not at school)
- ☐ elsewhere during free-time
- ☐ other place, where ? ____________

**33 What kind of injuries were caused by this accident?**

____________________

____________________

____________________

____________________

**34 Did you have to stay away from school, work or hobbies because of this accident?**

- ☐ no
- ☐ yes, for how long?
  - ☐ part of a day
  - ☐ a full day
  - ☐ 2-3 days
  - ☐ over 3 days

**35 Had you used alcohol at the time of accident?**

- ☐ no
- ☐ yes

## VIOLENCE

**36 During the past MONTH, have you been in a fight or an object of violence?**

- ☐ no (proceed to Question 43)
- ☐ yes, how many times?
  - ☐ once
  - ☐ twice
  - ☐ 3 times or more

**When you answer Questions 37-42, think of the LATEST fight or violent situation in which you were involved.**

**37 With whom did you fight or who treated you with violence? (tick as many alternatives as necessary)**

- ☐ complete stranger
- ☐ parent (or step-parent)
- ☐ sister or brother
- ☐ girlfriend/boyfriend
- ☐ friend
- ☐ other person I know (e.g. classmate)
- ☐ somebody else, who?____________

38 **Where did the fight or act of violence take place?**

- ☐ at home
- ☐ at school
- ☐ on the way to school
- ☐ in connection with sports activities (not at school)
- ☐ elsewhere in my free-time
- ☐ other place, where? ________________

39 **Did the fight or act of violence cause you physical injuries?**

- ☐ no
- ☐ yes, what kind?________________

________________________

________________________

________________________

40 **Did you have to stay away from school, work or hobbies because of the fight or act of violence?**

- ☐ no
- ☐ yes, for how long?
  - ☐ part of a day
  - ☐ one full day
  - ☐ 2-3 days
  - ☐ more than 3 days

41 **Had you used alcohol at the time of fight or violence?**

- ☐ no
- ☐ yes

42 **Had another participant used alcohol?**

- ☐ no
- ☐ yes
- ☐ I don't know

## SMOKING

43 **Have you ever tried smoking?**

- ☐ no (you may proceed to Question 52)
- ☐ yes

44 **How many cigarettes, cigars or pipefuls of tobacco have you smoked altogether?**

- ☐ none (proceed to Question 52)
- ☐ only one (proceed to Question 52)
- ☐ approximately 2 - 50
- ☐ more than 50

45 **When did you last smoke a cigarette, cigar or a pipeful of tobacco?**

- ☐ yesterday or today
- ☐ 2 - 4 days ago
- ☐ about a week ago
- ☐ about 2 weeks to 2 months ago
- ☐ about 2 - 6 months ago
- ☐ more than 6 months ago (proceed to Question 52)

46 **Which of the following alternatives best describes your PRESENT SMOKING habit?**

- ☐ I smoke once a day or more often
- ☐ I smoke once a week or more often, but not daily
- ☐ I smoke less often than once a week
- ☐ I do not smoke at the moment or I have stopped smoking

47 **How much do you smoke a day at present or how much did you smoke before you stopped? Please put a dash if you smoke less than once a day. Please answer every item.**

Manufactured cigarettes ____ a day

Hand-rolled cigaretts ____ a day

Cigars or pipe ____ times a day

48 **Have you bought any tobacco during the past MONTH for yourself?**

- ☐ no
- ☐ yes, WHERE? (answer all items)

| | No | Yes |
|---|---|---|
| Grocery store | ☐ | ☐ |
| Kiosk | ☐ | ☐ |
| Peers | ☐ | ☐ |
| Service station | ☐ | ☐ |
| Bar | ☐ | ☐ |
| Vending machine | ☐ | ☐ |
| Other | ☐ | ☐ |

49 **Has someone else (e.g. peers, sister/brother, parents) bought any tobacco for you during the past MONTH with your money?**

- ☐ no
- ☐ yes, once
- ☐ yes, several times

50 **Have you got tobacco during the past MONTH from elsewhere? Answer all items.**

| | No | Yes |
|---|---|---|
| Father or mother | ☐ | ☐ |
| Other adults | ☐ | ☐ |
| Sisters/brothers | ☐ | ☐ |
| Peers | ☐ | ☐ |
| I took some at home | ☐ | ☐ |
| Other, where? | ☐ | ☐ |

______________________

51 **How often do you smoke on your way to school or at school? Answer each item**

| | Never | Now and then | Every day |
|---|---|---|---|
| On the way to school | ☐ | ☐ | ☐ |
| At school, in school grounds | ☐ | ☐ | ☐ |
| Near school during school hours | ☐ | ☐ | ☐ |

## ALL ANSWER

52 **Have you ever tried snuff? How many times in your lifetime?**

- ☐ I have never tried
- ☐ I have tried once
- ☐ I have used snuff 2-50 times
- ☐ I have used snuff more than 50 times

53 **Do you use snuff at present?**

- ☐ not at all
- ☐ every now and then
- ☐ once a day or more often

54 **Have you bought tobacco during the past MONTH for your peers with their money?**

- ☐ no
- ☐ I have, once
- ☐ several times

55 **Do you know someone among your peers who within the past YEAR has tried drugs (hashish, paint thinners or other inhalants, medicines with intoxicating effect, or other similar substances)?**

- ☐ I don't know any young people
- ☐ I know one young person
- ☐ I know 2-5 young persons
- ☐ I know more than 5 young persons

56 **During the past YEAR, have you been offered any drugs in FINLAND?**

- ☐ no
- ☐ yes, who offered?
  - ☐ friends or peers
  - ☐ strangers

57 **Has your mother ever smoked tobacco during your lifetime?**

- ☐ she has never smoked
- ☐ yes, but she has stopped
- ☐ she smokes at present
- ☐ I have no mother or don't know

58 **Has your father ever smoked tobacco during your lifetime?**

- ☐ he has never smoked
- ☐ yes, but he has stopped
- ☐ he smokes at present
- ☐ I have no father or don't know

## EXERCISE AND SLEEP

**59 How often do you take sports or physical activities in your free time?**

A Training programmes, competitions or matches organized by a SPORTS CLUB

- ☐ never
- ☐ less than once a month
- ☐ 1 - 2 times a month
- ☐ about once a week
- ☐ 2 - 3 times a week
- ☐ 4 - 5 times a week
- ☐ approximately every day

B OTHER FREE TIME ACTIVITIES?

- ☐ not at all
- ☐ less than once a month
- ☐ 1 - 2 times a month
- ☐ about once a week
- ☐ 2 - 3 times a week
- ☐ 4 - 5 times a week
- ☐ approximately every day

**60 Which of the following alternatives best describes your sport habits? USUALLY I do sports or exercise so much that**

- ☐ I do not get out of breath or sweat
- ☐ I get out of breath and sweat a little
- ☐ I get out of breath and sweat to some extent
- ☐ I get out of breath and sweat a lot
- ☐ I have no physical activities in my free time

**61 How much do you walk during a day on the average?**

- ☐ I hardly walk at all
- ☐ about half an hour
- ☐ about one hour
- ☐ about 2 hours
- ☐ about 3 hours or more

**62 How many hours on the average do you sit daily at your desk, reading, writing, using a computer, or doing other such work?**

A ON WEEKDAYS OUTSIDE SCHOOL

- ☐ not at all
- ☐ about half an hour
- ☐ about one hour
- ☐ about 2 hours
- ☐ about 3 hours or more

B ON WEEKENDS

- ☐ not at all
- ☐ about half an hour
- ☐ about one hour
- ☐ about 2 hours
- ☐ about 3 hours or more

**63 Do you usually go to bed at a certain time?**

- ☐ very regularly
- ☐ fairly regularly
- ☐ rather irregularly
- ☐ very irregularly

**64 At what time do you usually go to bed on school or work days?**

- ☐ at about 9 pm or earlier
- ☐ about 9.30 pm
- ☐ about 10 pm
- ☐ about 10.30 pm
- ☐ about 11 pm
- ☐ about 11.30 pm
- ☐ about midnight
- ☐ about 00.30 am

**65 At what time do you usually wake up on school or work days?**

- ☐ about 6 am or earlier
- ☐ about 6.30 am
- ☐ about 7 am
- ☐ about 7.30 am
- ☐ about 8 am
- ☐ about 8.30 am or later

**66 Do you usually feel refreshed and energetic when you wake up in the**

LIQUOR STORE?

☐ no

☐ yes

74 **During the past SIX MONTHS, have you had any alcohol in a LICENSED RESTAURANT?**

☐ no

☐ yes

75 **During the past SIX MONTHS, have you purchased medium beer at a GROCERY STORE?**

☐ no

☐ yes

76 **During the past SIX MONTHS, have you had medium beer in a BAR or PUB, or CAFETERIA?**

☐ no

☐ yes

## DIET

77 **Are you on a special diet at present?**

☐ no (proceed to Question 81)

☐ yes

78 **What kind of diet is it? Please answer all items.**

| | Yes | No |
|---|---|---|
| I avoid certain foods because of allergy | ☐ | ☐ |
| Low-lactose or non-lactose | ☐ | ☐ |
| Diet for diabetics | ☐ | ☐ |
| Diet for sport or body-building purposes | ☐ | ☐ |
| Weight losing | ☐ | ☐ |
| Vegetarian | ☐ | ☐ |
| Other, what?_______________ | ☐ | ☐ |

79 **What is the reason that you wish to follow a special diet?**

_______________________________

_______________________________

_______________________________

80 **If you are on a vegetarian diet, does it include (answer all items)**

| | Yes | No |
|---|---|---|
| only vegetables and other plant products | ☐ | ☐ |
| also milk | ☐ | ☐ |
| also eggs | ☐ | ☐ |
| also fish or poultry | ☐ | ☐ |

## ALL ANSWER

81 **How much money on the average can you spend in a WEEK (not including expenses for housing, food and clothes)**

☐ less than 7 marks

☐ 8 - 14 marks

☐ 15 - 24 marks

☐ 25 - 39 marks

☐ 40 - 59 marks

☐ 60 - 100 marks

☐ 101 - 200 marks

☐ 201 - 400 marks

☐ over 400 marks

82 **What is your mother's current employment situation?**

☐ work outside home

☐ work at home

☐ unemployed or on temporary lay-off

☐ retired or on a long sick-leave

83 **What is your father's current employment situation?**

☐ work outside home

☐ work at home

☐ unemployed or on temporary lay-off

☐ retired or on a long sick-leave

MORNING?

☐ often or every morning
☐ quite often
☐ sometimes
☐ seldom or never

67 **Have you felt tired in the DAYTIME in the past MONTH?**

☐ not at all
☐ less than once a week
☐ on 1-2 days a week
☐ on 3-5 days a week
☐ daily or almost daily

## USE OF ALCOHOL

68 **How old were you when you had at least one glass of**

BEER? _________ years old
☐ I have never had a beer

LONG DRINKS?_________ years old
☐ I have never had Long Drinks

WINE? __________ years old
☐ I have never had wine

SPIRITS? _________ years old
☐ I have never had spirits or liquors

69 **How often do you use alcohol altogether? Try to include all occasions, even when you had only a very small amount, e.g. half a bottle of medium beer or a small glass of wine.**

☐ daily
☐ a couple of times a week
☐ once a week
☐ a couple of times a month
☐ about once a month
☐ about once in every two months
☐ 3 - 4 times in a year
☐ once in a year or less
☐ I do not use alcohol (proceed to Question 73)

70 **And how often do you use alcohol to get REALLY DRUNK?**

☐ once a week or more often
☐ about once or twice every month
☐ less often
☐ never

71 **The last time you had some alcohol, how would you describe yourself? were you**

☐ quite sober
☐ slightly drunk
☐ very drunk
☐ so drunk that I passed out

72 **Think of the last time you had alcohol, WHAT DID YOU DRINK and HOW MUCH? (If you were drinking with friends, say how much you personally drank.)**

______________________________
______________________________
______________________________
______________________________
______________________________
______________________________

## ALL ANSWER

73 **During the past SIX MONTHS, have you purchased any alcohol at a STATE**

**84 Do your parents know most of your friends?**

- ☐ yes, they both know
- ☐ only my father knows
- ☐ only my mother knows
- ☐ neither of them knows

**85 Do your parents know where you spend your Friday and Saturday evenings?**

- ☐ yes, always
- ☐ yes, sometimes
- ☐ mostly they do not know

**86 How easy or difficult is it for you to talk to the following persons about things that really bother you?**

FATHER
- ☐ very easy
- ☐ easy
- ☐ difficult
- ☐ very difficult
- ☐ I have no father

MOTHER
- ☐ very easy
- ☐ easy
- ☐ difficult
- ☐ very difficult
- ☐ I have no mother

FRIENDS
- ☐ very easy
- ☐ easy
- ☐ difficult
- ☐ very difficult
- ☐ I have no friends

## FOR BOYS ONLY (girls proceed to Question 88)

**87 How old were you when you had your first ejaculation?**

- ☐ I have not had an ejaculation yet
- ☐ 10-yrs or younger
- ☐ 11-yrs
- ☐ 12-yrs
- ☐ 13-yrs
- ☐ 14-yrs
- ☐ 15-yrs or older

## FOR GIRLS ONLY (boys proceed to Question 92)

**88 How old were you when you had your first menstruation?**

- ☐ I have not had a menstruation yet
- ☐ 10-yrs or younger
- ☐ 11-yrs
- ☐ 12-yrs
- ☐ 13-yrs
- ☐ 14-yrs
- ☐ 15-yrs or older

**89 Have you ever used contraceptive pills?**

- ☐ no
- ☐ yes

**90 Are you taking contraceptive pills at present?**

- ☐ no
- ☐ yes

**91 Have you ever used post-intercourse (morning-after) contraception?**

- ☐ I don't know what is post-intercourse contraception
- ☐ no
- ☐ yes, how many times?
  - ☐ once
  - ☐ twice
  - ☐ 3 or more times

## STATEMENTS (all answer)

**92 Reasonable use of alcoholic beverages is part of ordinary life.**

- ☐ I definitely agree
- ☐ I agree to some extent
- ☐ difficult to say
- ☐ I slightly disagree
- ☐ I definitely disagree

**93 It is important that people can sometimes get drunk in order to relax and speak out their minds.**

- ☐ I definitely agree
- ☐ I agree to some extent
- ☐ difficult to say
- ☐ I slightly disagree
- ☐ I definitely disagree

**94 The adverse effects of smoking are over-emphasized these days.**

- ☐ I definitely agree
- ☐ I agree to some extent
- ☐ difficult to say
- ☐ I slightly disagree
- ☐ I definitely disagree

**95 Smoking is everybody's own business.**

- ☐ I definitely agree
- ☐ I agree to some extent
- ☐ difficult to say
- ☐ I slightly disagree
- ☐ I definitely disagree

**96 It is a good thing that selling tobacco to persons under 18 is illegal.**

- ☐ I definitely agree
- ☐ I agree to some extent
- ☐ difficult to say
- ☐ I slightly disagree
- ☐ I definitely disagree

**97 Non-smoking areas must be available in cafeterias, pubs and restaurants for non-smokers.**

- ☐ I definitely agree
- ☐ I agree to some extent
- ☐ difficult to say
- ☐ I slightly disagree
- ☐ I definitely disagree

*We thank you for your answers!*
