# Supplementary figures and images for: Changes in self-rated health and its association with social determinants – repeated cross-sectional surveys among Finnish adolescents from 1981 to 2025
Source: BMC Public Health. 2026 Mar 18;26:1375. doi: 10.1186/s12889-026-27022-y (PMC13112845; doi:10.1186/s12889-026-27022-y)

**Supplement 2.** The proportions in self-rated health over the years


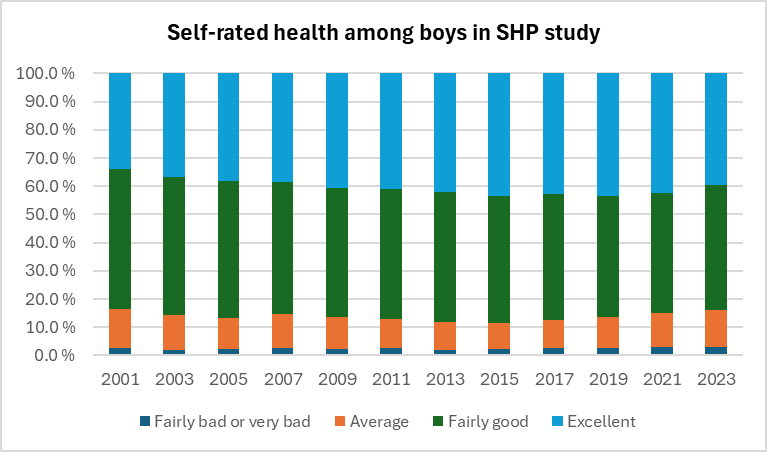


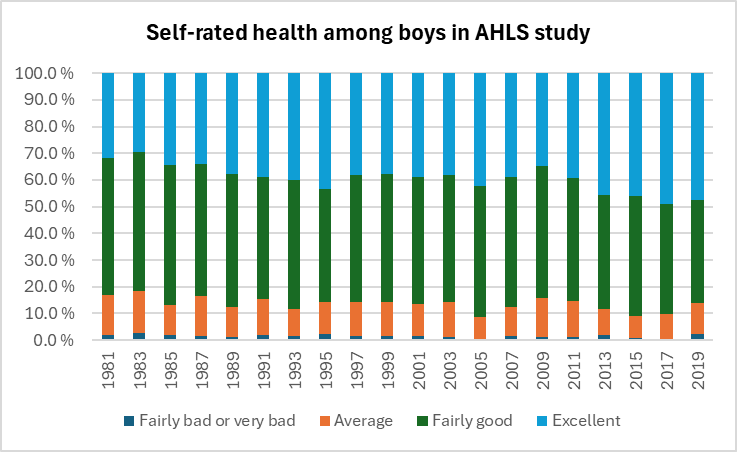


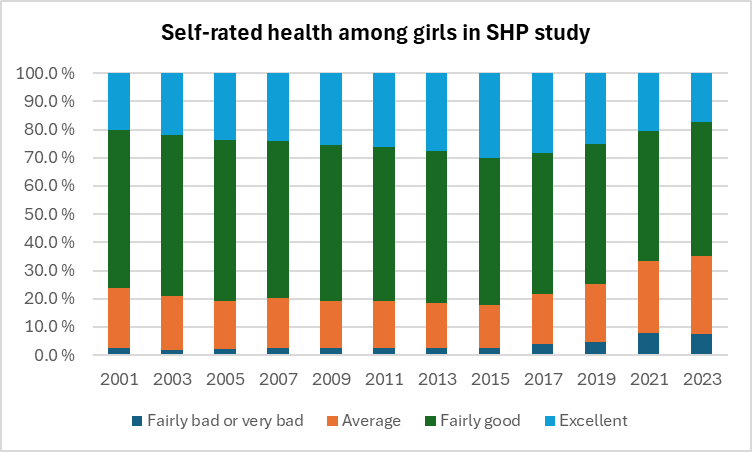


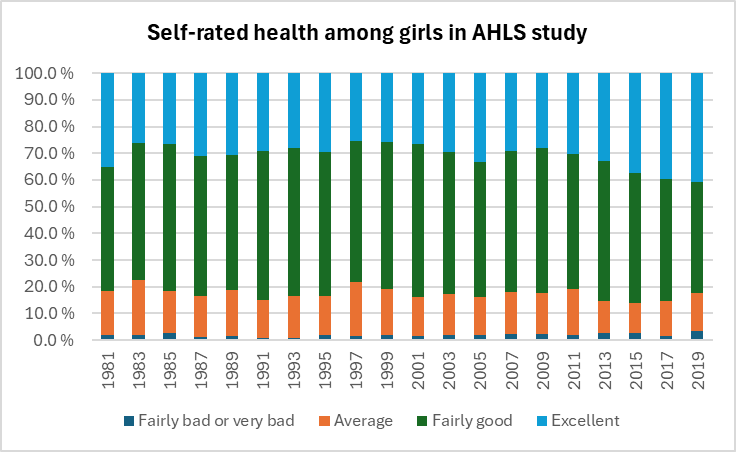

Supplement: Supplementary file 2 — Supplementary Material 2 [file 12889_2026_27022_MOESM2_ESM.docx]
