# Supplementary material for: Changes in self-rated health and its association with social determinants – repeated cross-sectional surveys among Finnish adolescents from 1981 to 2025
Source: BMC Public Health. 2026 Mar 18;26:1375. doi: 10.1186/s12889-026-27022-y (PMC13112845; doi:10.1186/s12889-026-27022-y)
Supplement: Supplementary file 3 — Supplementary Material 3 [file 12889_2026_27022_MOESM3_ESM.docx]

**Supplement 3.** Marginal effects for each explanatory variable according to study year among boys in SHP study

|  | **Parent’s education middle or low** | | | **Family structure (2000–2023)** | | | **Family structure (2013–2023)** | | | | | | **Smoking parent(s)** | | |
| --- | --- | --- | --- | --- | --- | --- | --- | --- | --- | --- | --- | --- | --- | --- | --- |
| **Year** | dy/dx | 99% CI | | Non-trational family dy/dx | 99% CI | | Non-trational family  dy/dx | 99% CI | | A shared residence dy/dx | 99% CI | | dy/dx | 99%CI | |
| 2001 | 0.023 | 0.185 | 0.027 | 0.076 | 0.071 | 0.082 |  |  |  |  |  |  | 0.064 | 0.059 | 0.068 |
| 2003 | 0.020 | 0.016 | 0.024 | 0.069 | 0.064 | 0.074 |  |  |  |  |  |  | 0.057 | 0.053 | 0.061 |
| 2005 | 0.019 | 0.015 | 0.022 | 0.064 | 0.059 | 0.069 |  |  |  |  |  |  | 0.053 | 0.049 | 0.057 |
| 2007 | 0.021 | 0.017 | 0.025 | 0.070 | 0.065 | 0.075 |  |  |  |  |  |  | 0.059 | 0.054 | 0.063 |
| 2009 | 0.019 | 0.016 | 0.023 | 0.066 | 0.061 | 0.071 |  |  |  |  |  |  | 0.055 | 0.051 | 0.060 |
| 2011 | 0.019 | 0.015 | 0.022 | 0.063 | 0.059 | 0.068 |  |  |  |  |  |  | 0.053 | 0.049 | 0.057 |
| 2013 | 0.018 | 0.014 | 0.021 | 0.062 | 0.057 | 0.066 | 0.079 | 0.072 | 0.087 | 0.020 | 0.013 | 0.028 | 0.052 | 0.048 | 0.056 |
| 2015 | 0.018 | 0.014 | 0.021 | 0.060 | 0.054 | 0.065 | 0.076 | 0.069 | 0.084 | 0.019 | 0.012 | 0.027 | 0.051 | 0.046 | 0.055 |
| 2017 | 0.019 | 0.015 | 0.022 | 0.065 | 0.060 | 0.071 | 0.084 | 0.076 | 0.093 | 0.022 | 0.013 | 0.030 | 0.055 | 0.050 | 0.059 |
| 2019 | 0.020 | 0.016 | 0.024 | 0.068 | 0.062 | 0.073 | 0.087 | 0.079 | 0.096 | 0.022 | 0.014 | 0.031 | 0.059 | 0.054 | 0.064 |
| 2021 | 0.022 | 0.018 | 0.026 | 0.074 | 0.068 | 0.080 | 0.095 | 0.086 | 0.104 | 0.025 | 0.015 | 0.034 | 0.065 | 0.060 | 0.070 |
| 2023 | 0.023 | 0.019 | 0.028 | 0.077 | 0.071 | 0.082 | 0.098 | 0.089 | 0.107 | 0.026 | 0.016 | 0.035 | 0.068 | 0.063 | 0.073 |
|  | **Poorer classroom atmosphere** | | | **Problems in relationships with teachers** | | | **Learning difficulty(ies)** | | |  |  |  |  |  |  |
| **Year** | dy/dx | 99% CI | | dy/dx | 99% CI | | dy/dx | 99% CI | |  |  |  |  |  |  |
| 2001 | 0.087 | 0.082 | 0.091 | 0.071 | 0.067 | 0.076 | 0.124 | 0.119 | 0.130 |  |  |  |  |  |  |
| 2003 | 0.078 | 0.074 | 0.082 | 0.064 | 0.060 | 0.068 | 0.111 | 0.106 | 0.115 |  |  |  |  |  |  |
| 2005 | 0.073 | 0.068 | 0.077 | 0.060 | 0.056 | 0.063 | 0.103 | 0.098 | 0.108 |  |  |  |  |  |  |
| 2007 | 0.079 | 0.074 | 0.083 | 0.065 | 0.061 | 0.069 | 0.113 | 0.108 | 0.117 |  |  |  |  |  |  |
| 2009 | 0.074 | 0.070 | 0.078 | 0.062 | 0.059 | 0.066 | 0.105 | 0.100 | 0.110 |  |  |  |  |  |  |
| 2011 | 0.071 | 0.067 | 0.075 | 0.059 | 0.056 | 0.063 | 0.101 | 0.097 | 0.106 |  |  |  |  |  |  |
| 2013 | 0.070 | 0.066 | 0.074 | 0.059 | 0.055 | 0.063 | 0.099 | 0.094 | 0.103 |  |  |  |  |  |  |
| 2015 | 0.069 | 0.064 | 0.074 | 0.059 | 0.054 | 0.063 | 0.100 | 0.094 | 0.106 |  |  |  |  |  |  |
| 2017 | 0.077 | 0.072 | 0.082 | 0.066 | 0.061 | 0.071 | 0.111 | 0.105 | 0.117 |  |  |  |  |  |  |
| 2019 | 0.083 | 0.078 | 0.088 | 0.071 | 0.066 | 0.075 | 0.118 | 0.112 | 0.124 |  |  |  |  |  |  |
| 2021 | 0.094 | 0.089 | 0.099 | 0.081 | 0.075 | 0.086 | 0.130 | 0.124 | 0.136 |  |  |  |  |  |  |
| 2023 | 0.095 | 0.089 | 0.100 | 0.082 | 0.077 | 0.087 | 0.134 | 0.128 | 0.140 |  |  |  |  |  |  |

Marginal effects for each explanatory variable according to study year among boys in AHLS study

|  |  | **Parents' education middle or low** | | | **Family structure (1981–2019)** | | | **Smoking parent(s)** | | | **Avarage or lower school performance** | | |
| --- | --- | --- | --- | --- | --- | --- | --- | --- | --- | --- | --- | --- | --- |
| **Year** |  | dy/dx | 95% CI | | Non-trational family dy/dx | 95% CI | | dy/dx | 95%CI | | dy/dx | 95% CI | |
| 1981 |  | 0.011 | -0.009 | 0.031 | 0.052 | 0.031 | 0.072 |  |  |  | 0.068 | 0.051 | 0.086 |
| 1983 |  | 0.012 | -0.010 | 0.034 | 0.055 | 0.033 | 0.077 |  |  |  | 0.073 | 0.055 | 0.092 |
| 1985 |  | 0.009 | -0.007 | 0.025 | 0.041 | 0.024 | 0.059 | 0.032 | 0.016 | 0.048 | 0.055 | 0.039 | 0.071 |
| 1987 |  | 0.011 | -0.009 | 0.031 | 0.050 | 0.030 | 0.070 | 0.037 | 0.019 | 0.056 | 0.069 | 0.051 | 0.087 |
| 1989 |  | 0.009 | -0.007 | 0.026 | 0.040 | 0.023 | 0.058 |  |  |  | 0.054 | 0.037 | 0.070 |
| 1991 |  | 0.010 | -0.008 | 0.029 | 0.048 | 0.028 | 0.067 | 0.036 | 0.018 | 0.054 | 0.064 | 0.046 | 0.082 |
| 1993 |  | 0.008 | -0.006 | 0.022 | 0.039 | 0.022 | 0.057 | 0.028 | 0.014 | 0.043 | 0.051 | 0.035 | 0.067 |
| 1995 |  | 0.010 | -0.008 | 0.027 | 0.045 | 0.026 | 0.064 | 0.034 | 0.017 | 0.051 | 0.059 | 0.042 | 0.077 |
| 1997 |  | 0.010 | -0.008 | 0.028 | 0.045 | 0.028 | 0.063 | 0.035 | 0.018 | 0.051 | 0.061 | 0.046 | 0.076 |
| 1999 |  | 0.010 | -0.008 | 0.027 | 0.044 | 0.027 | 0.062 | 0.034 | 0.018 | 0.051 | 0.059 | 0.045 | 0.073 |
| 2001 |  | 0.009 | -0.007 | 0.026 | 0.043 | 0.027 | 0.060 | 0.033 | 0.017 | 0.049 | 0.059 | 0.045 | 0.073 |
| 2003 |  | 0.010 | -0.008 | 0.027 | 0.044 | 0.027 | 0.061 |  |  |  | 0.061 | 0.047 | 0.075 |
| 2005 |  | 0.006 | -0.005 | 0.017 | 0.029 | 0.017 | 0.041 | 0.023 | 0.011 | 0.034 | 0.038 | 0.028 | 0.048 |
| 2007 |  | 0.009 | -0.007 | 0.024 | 0.040 | 0.024 | 0.056 | 0.030 | 0.015 | 0.045 | 0.054 | 0.041 | 0.068 |
| 2009 |  | 0.011 | -0.008 | 0.029 | 0.049 | 0.030 | 0.068 |  |  |  | 0.067 | 0.051 | 0.083 |
| 2011 |  | 0.010 | -0.008 | 0.028 | 0.046 | 0.028 | 0.065 |  |  |  | 0.063 | 0.047 | 0.079 |
| 2013 |  | 0.008 | -0.006 | 0.022 | 0.038 | 0.022 | 0.055 | 0.030 | 0.014 | 0.046 | 0.052 | 0.036 | 0.068 |
| 2015 |  | 0.007 | -0.005 | 0.018 | 0.031 | 0.018 | 0.044 | 0.024 | 0.012 | 0.036 | 0.041 | 0.030 | 0.053 |
| 2017 |  | 0.007 | -0.006 | 0.019 | 0.033 | 0.019 | 0.047 | 0.025 | 0.012 | 0.039 | 0.045 | 0.031 | 0.059 |
| 2019 |  | 0.010 | -0.008 | 0.027 | 0.043 | 0.026 | 0.061 | 0.035 | 0.017 | 0.053 | 0.059 | 0.043 | 0.076 |
